# Motor improvements enabled by spinal cord stimulation combined with physical training after spinal cord injury: review of experimental evidence in animals and humans

**DOI:** 10.1186/s42234-021-00077-5

**Published:** 2021-10-28

**Authors:** Ismael Seáñez, Marco Capogrosso

**Affiliations:** 1 Biomedical Engineering, Washington University in St. Louis, St. Louis, USA; 2 Neurosurgery, Washington University School of Medicine in St. Louis, St. Louis, USA; 3 Neurological Surgery, University of Pittsburgh, Pittsburgh, USA; 4 Department of Physical Medicine and Rehabilitation, Rehab and Neural Engineering Labs, University of Pittsburgh, Pittsburgh, USA; 5 Department of Bioengineering, University of Pittsburgh, Pittsburgh, USA

## Abstract

Electrical spinal cord stimulation (SCS) has been gaining momentum as a potential therapy for motor paralysis in consequence of spinal cord injury (SCI). Specifically, recent studies combining SCS with activity-based training have reported unprecedented improvements in motor function in people with chronic SCI that persist even without stimulation. In this work, we first provide an overview of the critical scientific advancements that have led to the current uses of SCS in neurorehabilitation: e.g. the understanding that SCS activates dormant spinal circuits below the lesion by recruiting large-to-medium diameter sensory afferents within the posterior roots. We discuss how this led to the standardization of implant position which resulted in consistent observations by independent clinical studies that SCS in combination with physical training promotes improvements in motor performance and neurorecovery. While all reported participants were able to move previously paralyzed limbs from day 1, recovery of more complex motor functions was gradual, and the timeframe for first observations was proportional to the task complexity. Interestingly, individuals with SCI classified as AIS B and C regained motor function in paralyzed joints even without stimulation, but not individuals with motor and sensory complete SCI (AIS A). Experiments in animal models of SCI investigating the potential mechanisms underpinning this neurorecovery suggest a synaptic reorganization of cortico-reticulo-spinal circuits that correlate with improvements in voluntary motor control. Future experiments in humans and animal models of paralysis will be critical to understand the potential and limits for functional improvements in people with different types, levels, timeframes, and severities of SCI.

## Background

Recent studies combining spinal cord stimulation (SCS) with activity-based training have reported lasting improvements in motor function that were historically thought impossible in the chronic stage of spinal cord injury (SCI). In 2018, three independent groups demonstrated, for the first time, that participants with chronic motor-complete SCI could achieve overground walking with SCS (Angeli et al. 2018; Gill et al. 2018; Wagner et al. 2018). Albeit only in a small number of patients, because of the size and consistency of the effects, these observations have been regarded as a giant step for SCI research (Moritz 2018) and a potential paradigm shift in rehabilitation strategies (Smith et al. 2019). In this work, we review the clinical evidence of long-term recovery induced by SCS after SCI and the experimental evidence on markers of neural plasticity that have been observed in animal and human studies.

### Spinal cord stimulation: from pain management to the first anecdotical observations of improvements in motor function

Epidural spinal cord stimulation has been a clinically approved technology for the treatment of neuropathic pain since the 1970s (Krames et al. 2009). Epidural SCS therapy requires the implantation of a silicone-based multi-electrode array in the epidural space between the spinal cord and the vertebral bone. The electrode array is then used to deliver continuous electrical pulses to the sensory afferents in the dorsal columns. The concept of SCS for pain management was first proposed after neurophysiological studies suggesting that it was possible to inhibit input from pain fibers into the spinal cord by the electrical stimulation of the large-diameter sensory fibers (Melzack and Wall 1965; Wall and Sweet 1967). Shealy and colleagues proved successful in 1967 in using subdural SCS to manage intractable chronic pain in cats (Shealy et al. 1967a) and immediately followed in one human patient with cancer (Shealy et al. 1967b). This paved the way for SCS to become a successful clinical therapy for pain management (Lempka and Patil 2018) with approximately 50,000 patients undergoing spinal cord stimulator implants each year (Sdrulla et al. 2018).

The relatively low invasiveness of the procedure to implant SCS leads quickly promoted the application of SCS in a variety of clinical conditions. In 1973, just a few years after the first human implants were performed, SCS-mediated improvements in motor function were unexpectedly observed: an individual with partial paralysis due to multiple sclerosis receiving SCS to treat chronic pain regained volitional control of her upper and lower extremities, facilitation of sitting, standing, and ambulation during stimulation (Cook and Weinstein 1973) – an improvement that had never been observed. These observations led to a cascade of investigational studies pioneering SCS applications for motor control that described improvements in motor, sensory, and bladder function by delivering SCS to participants with a wide variety of motor disorders (Dooley and Sharkey 1977; Siegfried et al. 1978; Siegfried et al. 1981; Waltz et al. 1981; Davis et al. 1981).

### Motor prosthetic effect of SCS after paralysis

In the early years of SCS, its potential therapeutic use was sought for a variety of conditions. The first applications of SCS on individuals with SCI focused primarily on spasticity management (Richardson and McLone 1978; Richardson et al. 1979; Barolat-Romana et al. 1985; Barolat et al. 1988). However, secondary effects on autonomic function including bowel control and sexual function, and motor capacity, were also observed (Richardson and McLone 1978; Richardson et al. 1979; Barolat-Romana et al. 1985; Barolat et al. 1988; Dimitrijevic et al. 1986). During these initial clinical applications, the ability of SCS to improve motor function after paralysis was not immediately understood. This may be attributable to evidence of functional improvements being purely empiric and occurring as additional observations during clinical treatment of pain. Indeed, when the first improvements in motor control were observed, they were initially attributed to reductions in spasticity enabled by SCS (Barolat et al. 1988). However, spasticity and motor deficits are two distinct phenotypes of SCI with different neural origins. Motor deficits do not emerge in consequence of velocity-dependent rigidity at the joints, instead, paralysis is driven by muscle weakness, e.g. the inability to activate spinal motoneurons (Li et al. 2012). Moreover, the ability of an individual with SCI to regain voluntary control of paralyzed muscles did not depend on changes in spasticity. Although spasticity remained present even after the stimulation was turned off, motor improvements temporarily enabled by SCS would be immediately lost (Barolat et al. 1988).

**Fig. 1:**
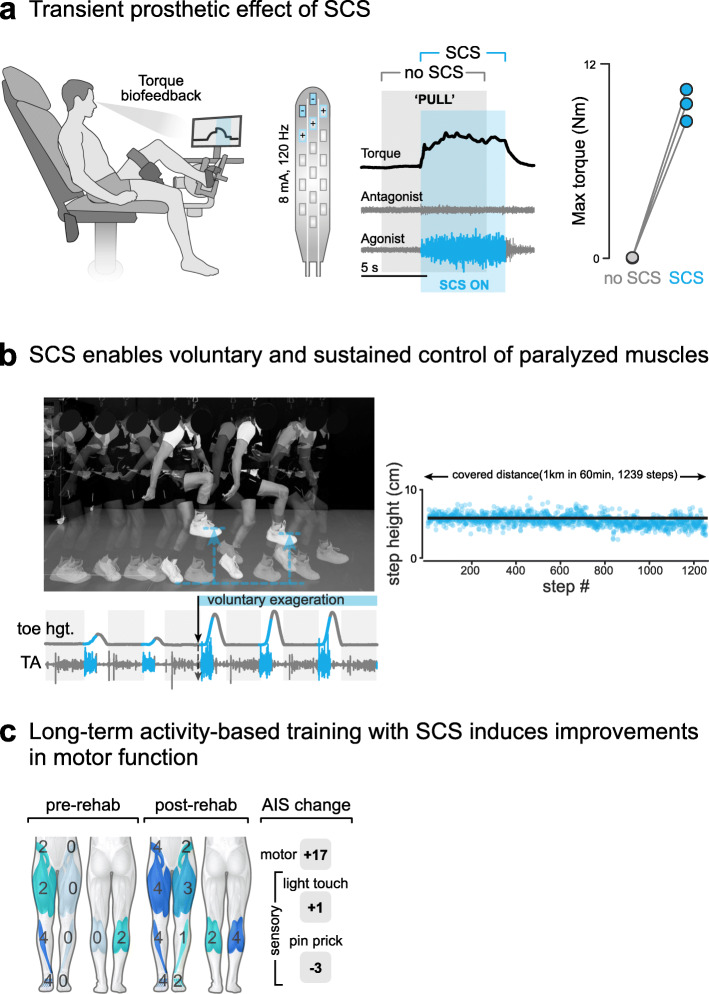
How can spinal cord stimulation lead to functional improvements in SCI? **a** SCS provides a prosthetic effect that enables activation of previously paralyzed muscles. **b** The prosthetic effect of SCS enables prolonged activation of paralyzed muscles in a physical therapy setting. **c** Long-term practice with activity-based training and SCS results in increases clinical measures of function in chronic SCI. Modified with permission from (Wagner et al. 2018)

The ability of SCS to immediately improve voluntary motor control constitutes the first breakthrough for applications of SCS in people with SCI. This can be considered a prosthetic effect: without any physical training, the activation of SCS enabled individuals to move previously paralyzed limbs and activate spinal motoneurons, thus overcoming muscle weakness (Fig. 1a). However, this effect was not lasting and would immediately disappear as soon as the stimulation was turned off. It is important to distinguish this concept from typical “therapy” as seen from the point of view of the rehabilitation therapist. Physical therapy leads to lasting changes in motor deficits, SCS alone did not. These observations led to a multitude of studies investigating the potential of this technology as a prosthesis capable of improving or enabling motor function after SCI in multiple settings (Siegfried et al. 1981; Davis et al. 1981; Dimitrijevic et al. 1986; Bamford and Davis 2019; Waltz 1997; Dimitrijevic et al. 1998) which largely replicated these results.

It is fair to ask why SCS did not become an established neuromodulation therapy for SCI if these results were so robust. As we mentioned, the mechanisms of SCS were not completely understood at this point; inferring mechanisms of action from clinical observations in humans led to a limited understanding. Human experiments in clinical settings allowed for a limited type and source of neural and biological signals to be recorded and evaluated. Additionally, implant locations were optimized for the treatment of pain, lacking consistency across participants and studies. Finally, stimulation parameters were set at the clinic, and low-intensity stimulation was constantly delivered throughout months without a training component to promote the continuous interaction of spared spinal circuits with voluntary descending drive. Together, these limitations constrained interpretations of the effects and therapeutic efficacy of SCS to restore motor function in SCI.

### Neural targets and optimal implant location

Studies following the initial observations from clinical studies aimed to understand the mechanisms of action of SCS. In particular, the specific neural elements that were primarily activated by SCS and how those led to recruitment of motoneurons were scientific questions of high interest. The combination of theoretical investigations employing the physics of electrical stimulation and the biophysics of neural membranes, as well as electrophysiology studies in humans and animals confirmed that epidural SCS directly recruits large-to-medium diameter proprioceptive and cutaneous afferents within the posterior roots of the spinal and the dorsal columns (Rattay et al. 2000; Ladenbauer et al. 2010; Capogrosso et al. 2013; Courtine et al. 2007a; Murg et al. 2000; Minassian et al. 2004; Minassian et al. 2007a). These sensory afferents convey excitatory post-synaptic potentials to the spinal motoneurons via mono- and poly-synaptic connections (Capogrosso et al. 2013; Minassian et al. 2007a; Minassian et al. 2016; Sayenko et al. 2014; Moraud et al. 2016; Greiner et al. 2021).

These studies led to two distinct conclusions: first, that the prosthetic effect of SCS can be explained by the increase of excitatory inputs to the spinal motoneurons that is provided by the synchronized excitatory postsynaptic potential volleys induced by each pulse of SCS via the sensory afferents; and second, that because of this mechanism, the optimal location of the electrode array coincides with the position of maximum likelihood of recruitment of the dorsal roots that innervate leg (or arm) muscles (Capogrosso et al. 2013; Greiner et al. 2021; Capogrosso et al. 2016; Capogrosso et al. 2018). For example, in the case of the legs, this corresponds to the T11-L1 vertebrae in humans (Angeli et al. 2018; Gill et al. 2018; Wagner et al. 2018; Dimitrijevic et al. 1986; Harkema et al. 2011; Angeli et al. 2014).

**Fig. 2:**
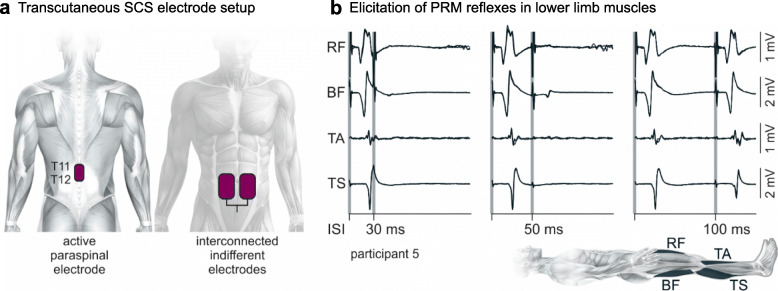
Post-stimulation depression of evoked responses confirms elicitation of posterior root-muscle (PRM) reflexes. **a** Electrode position for elicitation of muscle responses via transcutaneous SCS. **b** Afferent stimulation is confirmed by the depression and partial recovery of the PRM reflex using a paired-pulse paradigm (Minassian et al. 2007b; Hofstoetter et al. 2018; Kagamihara et al. 1998; Hofstoetter et al. 2020). Exemplary electromyographic responses of the leg muscles when the time between stimuli is set to 30, 50, and 100 ms. Responses to the second stimulus are completely eliminated at interstimulus intervals of 30 ms and partially recover at 100 ms (Hofstoetter et al. 2019). Modified with permission from (Hofstoetter et al. 2021)

On the validity of the first point, it is certainly possible that other mechanisms may also contribute to the prosthetic effect on volitional motor control. For example, the generation of plateau potentials in the motoneurons (Kiehn and Eken 1997; Heckman et al. 2005) polysynaptic spinal reflexes (Pinter et al. 2000; Hofstoetter et al. 2015a), as well as specialized spinal networks that control synergistic components of locomotor movements (Minassian et al. 2017; Danner et al. 2015) (Fig. 2). However, the understanding that SCS recruits mostly the dorsal roots, can be reliably used in first approximation to guide implant procedures and obtain robust and replicable results across participants (Angeli et al. 2018; Gill et al. 2018; Wagner et al. 2018; Capogrosso et al. 2018; Lu et al. 2016).

The second implication in the optimal location of the epidural array enabled standardization of practice which allowed to achieve robust results that have been reproduced by multiple independent clinical studies around the world (Angeli et al. 2018; Gill et al. 2018; Wagner et al. 2018; Sayenko et al. 2014).

### SCS-mediated neurorecovery in humans with SCI

Standardization of practice enabled interventional clinical trials to study whether the delivery of SCS in combination with physical training could improve functional outcomes in individuals with SCI. This concept was first explored in 2 individuals with incomplete SCI classified as American Spinal Injury Association Impairment Scale (AIS) C who improved their ability for treadmill and overground walking after training with SCS in addition to weight-assisted training, further than what could be achieved by weight-assisted training alone (Herman et al. 2002; Carhart et al. 2004). Subsequent studies in rodent models of SCI further solidified this concept by demonstrating the regained ability to walk and sustain full body weight when SCS was combined with locomotor training (Fig. 1b) in larger groups (Ichiyama et al. 2008; Courtine et al. 2009; van den Brand et al. 2012; Courtine et al. 2008). Recently, at least three independent clinical trials have reported improved functional outcomes besides walking, with and without stimulation, after intense physical therapy in combination with SCS (Fig. 1c). The results are summarized in Table 1.

**Table 1:** Functional outcomes after SCS and activity-based training

Functional outcomes	Reported with SCS on	Reported in	Time of first observation after surgery (SCS on)	Reported with SCS off	Reported in
**Voluntary muscle contraction**	8/8 participants	Angeli et al. 2018; Gill et al. 2018; Wagner et al. 2018	Day 1	4/8 participants AIS B/C/D	Angeli et al. 2018; Wagner et al. 2018
**Single joint movements of paralyzed joints**	8/8 participants	Angeli et al. 2018; Gill et al. 2018; Wagner et al. 2018	Day 1	2/8 participants AIS C/D	Wagner et al. 2018
**Standing**	8/8 participants	Angeli et al. 2018; Gill et al. 2018; Wagner et al. 2018	Week 1	4/8 participants AIS C/D	Angeli et al. 2018; Wagner et al. 2018
**Treadmill walking**	8/8 participants	Angeli et al. 2018; Gill et al. 2018; Wagner et al. 2018	3/8 > Week 2 5/8 > Week 10	n.r.	
**Overground walking**	6/8 participants (with assistance e.g. walker)	Angeli et al. 2018; Gill et al. 2018; Wagner et al. 2018	> Week 20	2/8 participants AIS C/D^a^ (with assistance e.g. walker)	Wagner et al. 2018

Included studies: Angeli et al. 2018 (NCT02339233), Gill et al. 2018 (NCT02592668), Wagner et al. 2018 (NCT02936453). Included participants (*N* = 8): 3 AIS A (motor and sensory complete), 2 AIS B (motor complete), 3 AIS C/D (motor incomplete)

^a^One participant in Wagner et al. 2018 achieved steps overground without additional support 3 months after rehabilitation (parallel bars present for safety but not touched)

The three reported studies achieved similar clinical outcomes albeit with different timings. This may be attributable to the efficiency of parameter optimization for optimal, patient-specific stimulation protocols (Angeli et al. 2018; Wagner et al. 2018; Formento et al. 2018). However, in terms of clinical and scientific outcomes, the results are remarkably consistent. First, all studies reported on the efficacy of SCS as a prosthetic intervention to enable movements of previously paralyzed joints since day 1 after implantation. Second, participants progressively acquired abilities of increasing complexity after the onset of SCS-assisted physical training focused on walking and standing. The ability to stand with SCS was reported in all participants 1 week after the onset of therapy, and participants were able to move their previously paralyzed legs while walking on a treadmill after the 2nd week. Twenty weeks after the onset of training, 6/8 participants were able to walk overground with a minimal level of support (walker, crutches, etc.) in addition to SCS. Perhaps of the highest importance, partial regaining of motor function was reported in participants even when the stimulation was off by the end of training. Interestingly, the 3/4 participants that did not achieve this goal were classified as having a motor and sensory complete SCI (AIS A), whereas the 4/8 participants that could activate their previously paralyzed muscles, even without SCS were classified as motor complete or incomplete (AIS B, C, and D). Two of them (AIS C and D) could also perform isolated single-joint movements, stand without external support, and walk with an assistive device, all without stimulation.

Taken together with the pioneering reports from Herman and colleagues (Herman et al. 2002; Carhart et al. 2004), these studies show that SCS in combination with physical training promotes neurorecovery and induces lasting changes enabling improvements in motor performance (with and without SCS) and rehabilitation outcomes. While larger studies are required to evaluate the overall effect size of SCS therapy for different SCI populations, motor improvements in the chronic stage of SCI are remarkable compared to previous reports showing no additional improvements in walking function when comparing locomotor training to bodyweight supported treadmill training with or without functional electrical stimulation or robotic-assisted locomotor training (Mehrholz et al. 2012).

### The need to understand mechanisms of recovery

**Fig. 3:**
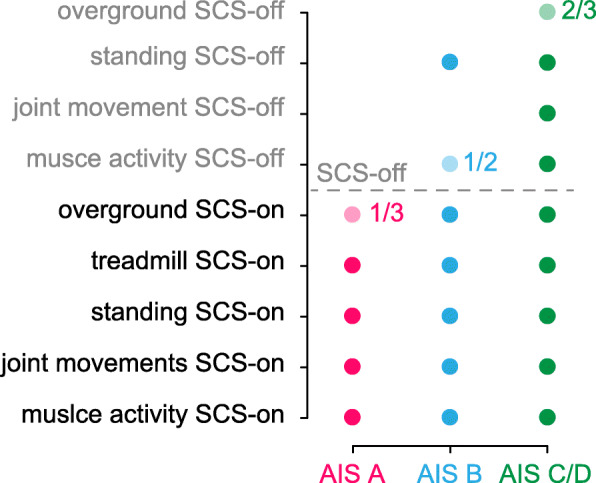
Functional improvements after long-term training with SCS and activity-based training. Summary of recovery outcomes extracted from Table 1 as a function of lesion severity assessed by AIS classification at study enrollment. Transparent circles indicate not all participants with that AIS classification achieved the outcome. Adjacent ratios indicate the partial number

While population size is too small to make definitive conclusions, results from the most recent studies (Table 1) seem to indicate that recovery outcomes correlate with lesion severity at study enrollment (Fig. 3). On one side this result is not surprising. Since SCS seems to amplify voluntary motor control (Fig. 1a), the capacity of an individual to achieve complex motor tasks must depend on the amount of residual supra-spinal inputs after the lesion. However, the apparent dependence of functional improvements on lesion severity also represents an opportunity to improve on current rehabilitation approaches that could maximize outcomes of SCS-enhanced physical therapy to improve rehabilitation outcomes for individuals with AIS A/B SCI. To this end, studies on the neural mechanisms that may explain neurorecovery induced by SCS and combined physical training are crucial. Having a better understanding of the contribution of specific supra-spinal inputs, as well as the timing and dynamics of neurorecovery may allow improvements in a priori patient selection, optimization of stimulation parameters, and personalized rehabilitation protocols that take advantage of patient-specific residual functions to improve and accelerate neurorecovery. Basic research on animal models of paralysis will remain a key component of this effort (Courtine et al. 2007b).

### Animal models of SCS-mediated neurorecovery

Experiments in animal models of SCI enabled investigations into the intrinsic neural mechanisms and contributors of SCS-mediated recovery. One of the first models explicitly designed to study voluntary motor control enabled by SCS was a double-hemisection rat model of SCI. Van den Brand and colleagues performed a left lateral over-hemisection at the thoracic vertebra (T7) and a right lateral hemisection below at T10 (van den Brand et al. 2012). This procedure completely interrupted supraspinal pathways while leaving a gap (from T8-T9) of intact spinal cord, mimicking maintenance of connections through the lesion even in individuals with clinically complete SCI (Sherwood et al. 1992; McKay et al. 2004). After the complete loss of hindlimb function from the SCI, epidural SCS combined with serotonin and dopamine receptor agonists promoted coordinated stepping on a treadmill – but not overground – with body-weight support as early as 7 days after injury. The combination of the prosthetic effect of SCS with long-term activity-based training focused on promoting voluntary movement by training on over-ground locomotion resulted in improvements in walked distance covered in a fixed amount of time and voluntarily mediated gait in stairs and obstacle avoidance.

Anatomical examinations with retrograde tract-tracing revealed an increase in the number of neurons in the intermediate and ventral laminate of the inter-lesion segments (from T8-T9) in trained rats that were active during walking, suggesting these neurons may play a pivotal role in restoring voluntary locomotion. Although the SCI interrupted 98% of corticospinal tract axons, overground-trained rats recovered ~ 45% or pre-lesion fiber density in the inter-lesion dorsolateral funiculus, bypassing the T7 over-hemisection, branching into the gray matter, and recrossing the midline to develop bouton-like swellings, suggesting regenerative sprouting (Steward et al. 2003) mediated by the combination of SCS with activity-based training and dopamine/serotonin agonists. An increase in the density of cortical projections was also found in various brainstem motor areas containing reticulospinal neurons that project spinally to serotonergic neurons: the vestibular nuclei, the entire reticular formation, and the pyramidal regions. Importantly, these improvements in motor function and neural plasticity were observed only for rats that were received overground training which promotes voluntary movements, but not on the ones that trained only on a treadmill that may instead trigger automated stepping behaviors (Sławińska et al. 2012a). Taken together, these results constitute anatomical evidence of a synaptic reorganization of residual projections that correlate with the ability of rats to regain supraspinal control of spinal circuits.

The double-hemisection rat model allows properly controlled experiments, a precise quantification of the SCI, and unambiguous conclusions about anatomical reorganization. However, SCI in humans results primarily from trauma (Center NSCIS 2021), resulting in high variations in damage to the spinal cord and residual pathways between individuals. Therefore, the mechanisms of SCS-mediated recovery after severe contusions remained largely debated and unexplored (Sławińska et al. 2012b; Wernig 2014). Asboth, Friedly, Beauparlant, and colleagues tackled this question by investigating the role of cortico-reticulo-spinal circuits in motor recovery in a rat model of contusion SCI (Asboth et al. 2018). Similar to rats with the double hemisection SCI, combined SCS and serotonergic and dopaminergic receptor agonists immediately restored treadmill stepping with body-weight support. After 9 weeks of 6 d per week training, rats regained weight-bearing locomotion, voluntary stepping on a staircase, and swimming across a pool of water without neuromodulation.

Optogenetic stimulation of pyramidal neurons projecting from the motor cortex to lumbar segments in mice with contusion SCI triggered weight-bearing locomotion during neuromodulation. In contrast, having only optogenetic stimulation or the electrochemical neuromodulation by turning the other one off was not sufficient to engage the paralyzed legs, suggesting that SCS enables the motor cortex to modulate locomotor movements of paralyzed legs. Viral tracing of the leg motor cortex revealed complete abolishment of corticospinal tract projections below the injury but spared connectivity with lumbar segments in a subset of projection neurons located mostly in the ventral gigantocellular reticular nuclei (vGi), a subregion of the medullary reticular formation. Silencing of glutamatergic neurons in the vGi blocked the cortical control of locomotion that had been previously enabled by optogenetic stimulation of the motor cortex and electrochemical stimulation of the spinal cord. Moreover, viral tracing and inactivation of these circuits in rats with contusion SCI revealed a reorganization triggered by neurorehabilitation with electrochemical SCS, and that regaining of volitional movements after training was contingent on these projections.

Several animal studies in rats (Ballermann and Fouad 2006; Zörner et al. 2014) and non-human primates (Zaaimi et al. 2012) have reported that supraspinal commands from the cortex can reach the spinal cord through brainstem pathways after SCI, and these have been shown to contribute towards recovery after SCI in humans (Baker and Perez 2017). In summary, animal models of SCI suggest that cortico-reticulo-spinal circuits may mediate the volitional control of paralyzed areas enabled by electrical stimulation of the spinal cord (Angeli et al. 2014; Gerasimenko et al. 2015), and activity-dependent reorganization of these circuits enabled by SCS may be a primary contribution towards the restoration of function without stimulation that has been observed in humans (Angeli et al. 2018; Gill et al. 2018; Wagner et al. 2018).

## Are motor improvements limited to individuals with spared descending neural fibers?

The combined results from the three main clinical studies included in this review suggest recovery outcomes are directly related to lesion severity (Fig. 3) which we believe is indicative of the link between SCS and the amount of spared descending neural fibers. Although studies with a greater number of individuals with different AIS grading scales (A, B, C, and D) are needed to validate these preliminary observations, we expect that this trend will be confirmed independently from inclusion criteria. Indeed, inclusion criteria were already considerably wide across the three studies, and represented a wide variety of participants ranging from motor complete to incomplete (Angeli et al., 2018: SCI above T10 and at least 1 year post-injury; Gill et al., 2018: SCI between C7-T10, AIS A or B, at least 2 years post-injury; Wagner et al., 2018: SCI above T10, AIS A, B, C, or D, at least 1 year post-injury). Moreover, by combining evidence from both early and recent studies SCS can be thought of as a technique that amplifies residual descending voluntary input while simultaneously supporting the excitatory drive to the motoneurons, thus enhancing motor function and strength (Minassian et al. 2016; Guiho et al. 2021). This unique combination enables the integration of voluntary inputs with corresponding, sustained motor outputs that can lead to the activity-dependent plasticity that has been observed in animal models of SCI and humans (Moraud et al. 2016; Formento et al. 2018).

While we believe that residual supra-spinal input may determine the upper-bounds for potential clinical outcomes, it is important to note that anatomical (Kakulas 1988) and neurophysiological evidence of residual descending white matter and voluntary input has been found in a majority of individuals clinically classified as having a complete SCI (Sherwood et al. 1992), and 10% of these individuals with discomplete SCI can generate traces of motor unit activity, yet not strongly enough to elicit a visible contraction or movement (McKay et al. 2004). Perhaps unsurprisingly, participants classified as having a “motor complete” SCI were able to recover voluntary movements with SCS as early as day 1 (Table 1).

To properly address the topic of responsiveness to SCS, future clinical studies should perform objective evaluations of the integrity of residual descending projections that go further than clinical function and impairment scores. In this case, initial quantifications of spared neural circuits could dictate the types of rehabilitation exercises that could maximize an individual’s potential for SCS-mediated neurorecovery. In addition, SCI research will greatly benefit from studies on large numbers of participants to determine other factors that may affect responsiveness to SCS, such as sex, race, genetics, and economic backgrounds and generate evidence-based knowledge that appropriately represents target populations to addresses their specific needs.

## What types of training activities are most likely to maximize recovery outcomes?

The studies presented in this work make clear that volitional effort from the participant is essential. When stimulation is provided alone, as done initially five decades ago, improvements in motor performance were not consistently observed. Similarly, other rehabilitation methods like robotic training are more effective when requiring volitional effort from the participants than when full assistance is provided such that stepping is produced regardless of participant effort (Field-Fote and Roach 2011; Lam et al. 2015).

In retrospect, this should not be surprising given the interpretation of SCS as potentiating residual descending inputs (Minassian et al. 2016; Guiho et al. 2021) and the assumption that these inputs are necessary to generate plasticity (Kakulas 1988; Dimitrijevic et al. 1984). However, unexplored questions concern the specific exercises that may or may not maximize outcomes. Functional improvements in motor function have been reported by combining SCS with different types of activity-based training, from walking to hand and arm function (Angeli et al. 2018; Gill et al. 2018; Wagner et al. 2018; Inanici et al. 2021). Training programs that incorporate multiple types of activities have the potential for improved outcomes compared to training on a single task (Yang et al. 2014). This is further supported by recent studies showing that the consolidation of learning – i.e. the long-term retention of acquired skills – is better achieved by practicing multiple diverse tasks than by focusing on a single task (Kantak et al. 2010; Kantak and Winstein 2012). Therefore, training programs should aim at maximizing volitional inputs while implementing diverse types of tasks.

Because some arm function is essential to use assistive devices like walkers and crutches in locomotor training, the inclusion of individuals with injuries above C5 in locomotor-type rehabilitation would present significant challenges. Nevertheless, novel rehabilitation strategies using wearable sensors (Seanez-Gonzalez et al. 2016; Seanez-Gonzalez et al. 2017; Pierella et al. 2017) could incorporate lower extremity motor functions into SCS and activity-based training paradigms without the need for the highly demanding locomotor training. Indeed, it is possible to significantly improve walking function in people with SCI even without gait-specific training (Zhou et al. 2018).

How should be exercises be diversified? We believe that the way to approach this problem is tightly connected to the diversity of function of the different components of brain-spinal projections. For example, corticospinal projections contribute to dexterous tasks in humans (Bunday et al. 2014; Perez and Rothwell 2015) while reticulospinal circuits may contribute to strength (Glover and Baker 2020) and may be critical to recovery after corticospinal tract lesions or SCI (Zaaimi et al. 2012; Baker and Perez 2017). Therefore, we believe that there should be sufficient diversity in rehabilitation exercises to promote circuit-specific plasticity via SCS. For example, an individual with residual corticospinal tract input may benefit from the training of dexterous tasks and distal movements (Welniarz et al. 2017; Sangari and Perez 2020; Perez et al. 2004), whereas residual reticulospinal connections may dictate training of postural balance, strength production, and bimanual tasks (Glover and Baker 2020; Prentice and Drew 2001; Maslovat et al. 2020).

## Translating high-intensity training to clinical rehabilitation

Improvements in motor function mediated by SCS have been observed after high-intensity rehabilitation protocols that require daily practice and months-length durations. In contrast, rehabilitation protocols covered by insurance in the early and intermediate phases of SCI are low intensity and short in duration. Moreover, clinical SCI rehabilitation protocols usually end when a patient reaches a plateau in performance (Behrman et al. 2006; Thuret et al. 2006), and individuals with chronic SCI rarely receive additional rehabilitation coverage. Additional controlled clinical trials that evaluate the effectiveness of training intensity on rehabilitation outcomes are warranted to justify insurance coverage for these types of neurorehabilitation strategies. In addition, innovations that reduce the number of experts needed for SCS-mediated training and reduce the need for daily commute to the clinic may improve access for those with SCI and accelerate the adoption of these neurotechnologies for low-cost, at-home neurorehabilitation.

## Non-invasive alternatives to epidural stimulation

Transcutaneous SCS was developed (Minassian et al. 2007b) as a non-invasive method to activate the same neural structures, via similar mechanisms, as epidural SCS (Ladenbauer et al. 2010; Danner et al. 2011; Hofstoetter et al. 2018). The low-threshold sites along proprioceptive fibers at the posterior rootlet-spinal cord interface (Rattay et al. 2000) make the recruitment of posterior roots by skin-surface electrodes possible (Ladenbauer et al. 2010). In this manner, transcutaneous SCS can be used to augment muscle activity through mono- and polysynaptic spinal reflexes (Minassian et al. 2007b; Hofstoetter et al. 2008; Dy et al. 2010) and enable functional movements during treadmill stepping in individuals with chronic, motor incomplete SCI (AIS D) (Minassian et al. 2010; Hofstoetter et al. 2013; Hofstoetter et al. 2015b). And although transcutaneous SCS suffers from low selectivity in muscle recruitment compared to epidural SCS (de Freitas et al. 2021), different electrode positions can improve preferential activation of rostro-caudal (Krenn et al. 2015) or lateral (Calvert et al. 2019) spinal networks. Moreover, long-term rehabilitation with SCS combined with activity-based training can improve standing and balance (Sayenko et al. 2019) and induce functional recovery in people with SCI (Gerasimenko et al. 2015; Gad et al. 2017). Notably, improvements in balance control and reduced dependence on external assistance were quantitatively similar to those seen with epidural SCS (Harkema et al. 2011; Rejc et al. 2015). Finally, recent studies by several groups further highlight the need to combine SCS therapy with activity-based training to enable consistent improvements in walking (Shapkova et al. 2020; Estes et al. 2021), sit-to-stand (Al’joboori et al. 2020), and hand and arm function (Inanici et al. 2021) in people with SCI.

## Enhanced potential for high-intensity rehabilitation by improving autonomic function

Severe injuries above T6 commonly lead to hemodynamic instability and cardiovascular dysfunction that cause repeated hypotensive episodes known as autonomic dysreflexia (Weaver et al. 2012). Although intense activity-based training in the early phases of SCI is crucial for functional recovery, these life-threatening hypotension episodes hinder activity-based rehabilitation programs for individuals with SCI (Ashley et al. 1993; Illman et al. 2000). Recent studies have shown that by engaging sympathetic circuitry, SCS can restore hemodynamic stability after SCI (Squair et al. 2021; Phillips et al. 2018; Harkema et al. 2018). By improving cardiovascular function with SCS, it may be possible to avoid hypotension episodes during early rehabilitation and increase training frequency and intensity towards improved neurorecovery (Harman et al. 2021; Ditterline et al. 2020).

## Overcoming limitations of SCS through multi-intervention approaches

Although SCS-facilitated therapies have shown unprecedented improvements in motor function that persist even without stimulation, increases in strength production, range of motion, and even clinical outcomes have not been shown to have a significant, positive impact on the quality of life of people with SCI. Therefore, rehabilitation strategies should be aligned to the health and life priorities of individuals with SCI (Simpson et al. 2012). Interventions that improve arm/hand function, mobility, bowel, bladder, and sexual function, will be crucial to enable the independence and well-being of people with SCI. Multi-intervention approaches that contain a rehabilitation component in combination with SCS are likely to improve clinical measures of function and independence in people with SCI to a higher level than SCS alone (Gomes-Osman et al. 2016). Future SCI interventions will likely involve the combination of SCS with therapies using pharmacological neuromodulation (Gad et al. 2017; Radhakrishna et al. 2017; Freyvert et al. 2018) to engage spinal circuits or neural stem cells (Grill et al. 1997; Lu et al. 2012; Kadoya et al. 2016) and antibodies (Kucher et al. 2018; Freund et al. 2007) to promote axonal regeneration and synapse formation in SCI.

## Conclusion

The prosthetic effect of SCS on motor function below the injury can enable the delivery of activity-based interventions that lead to unprecedented functional improvements in the chronic stage of paralysis (Angeli et al. 2018; Gill et al. 2018; Wagner et al. 2018). However, whether these improvements are the result of the larger set of exercises that participants with SCI can execute with the support of SCS, or whether electrical stimulation of spinal circuits can somehow promote plasticity via the creation of a plasticity-permissive environment remains an important open question (Benavides et al. 2020; Rejc et al. 2020). While direct recordings and stimulation of cortico-reticulo-spinal circuits with viral tracing and optogenetics are not currently possible in humans, the use of transcranial magnetic stimulation (Perez et al. 2004; Benavides et al. 2020; Sangari and Perez 2019), startling responses (Baker and Perez 2017; Sangari and Perez 2020; Sangari and Perez 2019), peripheral nerve stimulation (Kumru et al. 2021), as well as functional (Weber et al. 2016) and structural (Smith et al. 2020; Smith et al. 2018) MRI, can provide a unique opportunity to evaluate the short- and long-term contributions of these neural circuits towards neurorecovery enabled by SCS and activity-based training. The continuous interaction between clinical trials and studies in animal models of paralysis will be critical to refine our understanding of the recovery capacity of the nervous system, ultimately leading to the development of optimized SCS-enabled rehabilitation strategies that accelerate neurorecovery.

## Data Availability

Not applicable.

## Abbreviations

SCS: spinal cord stimulation
SCI: spinal cord injury
AIS: American Spinal Injury Association Impairment Scale
vGi: ventral gigantocellular reticular nuclei
